# Intra- and inter-nucleosomal interactions of the histone H4 tail revealed with a human nucleosome core particle with genetically-incorporated H4 tetra-acetylation

**DOI:** 10.1038/srep17204

**Published:** 2015-11-26

**Authors:** Masatoshi Wakamori, Yoshifumi Fujii, Noriyuki Suka, Mikako Shirouzu, Kensaku Sakamoto, Takashi Umehara, Shigeyuki Yokoyama

**Affiliations:** 1 RIKEN Systems and Structural Biology Center, 1-7-22 Suehiro-cho, Tsurumi, Yokohama 230-0045, Japan; 2 RIKEN Center for Life Science Technologies, 1-7-22 Suehiro-cho, Tsurumi, Yokohama 230-0045, Japan; 3 RIKEN Structural Biology Laboratory, 1-7-22 Suehiro-cho, Tsurumi, Yokohama 230-0045, Japan; 4 School of Science and Engineering, Meisei University, 2-1-1 Hodokubo, Hino, Tokyo 191-8506, Japan; 5 PRESTO, Japan Science and Technology Agency (JST), 4-1-8 Honcho, Kawaguchi, Saitama 332-0012, Japan

## Abstract

Post-translational modifications (PTMs) of histones, such as lysine acetylation of the N-terminal tails, play crucial roles in controlling gene expression. Due to the difficulty in reconstituting site-specifically acetylated nucleosomes with crystallization quality, structural analyses of histone acetylation are currently performed using synthesized tail peptides. Through engineering of the genetic code, translation termination, and cell-free protein synthesis, we reconstituted human H4-mono- to tetra-acetylated nucleosome core particles (NCPs), and solved the crystal structures of the H4-K5/K8/K12/K16-tetra-acetylated NCP and unmodified NCP at 2.4 Å and 2.2 Å resolutions, respectively. The structure of the H4-tetra-acetylated NCP resembled that of the unmodified NCP, and the DNA wrapped the histone octamer as precisely as in the unmodified NCP. However, the *B*-factors were significantly increased for the peripheral DNAs near the N-terminal tail of the intra- or inter-nucleosomal H4. In contrast, the *B*-factors were negligibly affected by the H4 tetra-acetylation in histone core residues, including those composing the acidic patch, and at H4-R23, which interacts with the acidic patch of the neighboring NCP. The present study revealed that the H4 tetra-acetylation impairs NCP self-association by changing the interactions of the H4 tail with DNA, and is the first demonstration of crystallization quality NCPs reconstituted with genuine PTMs.

The fundamental unit of chromatin compaction is the nucleosome. The nucleosome core particle (NCP) is formed by wrapping the histone octamer, comprising two copies of the four core histones H2A, H2B, H3 and H4, with the superhelices of a 145–147 bp DNA duplex^1^,^2^. Each core histone is composed of the N-terminal tail region and the core region^1^,^2^. The core regions of the four core histones adopt histone folds, which mainly contribute to the formation of the histone octamer structure. In contrast, the N-terminal tails of the core histones, which are rich in positively-charged lysine and arginine residues, protrude through the DNA superhelices^1^. Since the determination of the 2.8 Å resolution structure of the *X. laevis* NCP^1^,^3^, many high-resolution NCP structures have been reported, including NCPs from different species such as human^4^, NCPs containing histone variants^5^,^6^,^7^, NCPs complexed with small compounds^8^,^9^, and NCPs complexed with chromatin-interactive peptides or proteins^10^,^11^,^12^. Furthermore, the 9 Å resolution crystal structure of a compact tetra-nucleosome^13^ and the 11 Å resolution cryogenic electron microscopy structures of 30 nm chromatin fibers have been reported^14^.

The N-terminal tails of the core histones are partially responsible for the structural integrity of the NCP. With their abundance of positively-charged residues, the tail domains increase the thermal stability of mono-nucleosomes^15^. The removal of the tail has relatively small effects on nucleosome stability and DNA wrapping ability, as compared to the stimulation by transcription factor binding^16^,^17^,^18^. For example, the deletion of residues S1 to A15 of the H4 N-terminal tail did not significantly affect the crystal structure of the NCP^19^. However, the deletion of the N-terminal tail of H2B enhances nucleosome sliding along DNA^20^, probably because the histone•DNA interactions are reduced, and the nucleosome is destabilized^17^,^19^,^20^. The N-terminal tails of the core histones perform additional functions required for the formation of higher-order chromatin structures, especially for cation-dependent folding and self-association at the levels from the secondary to tertiary structures of chromatin^21^,^22^,^23^,^24^,^25^. Furthermore, the N-terminal tail of H4 is crucial for the compact folding into chromatin fibers^26^,^27^. This function presumably occurs through the interaction between the H4 N-terminal tail and the acidic patch of the neighboring nucleosome, which was detected in the crystal packing of the mono-NCP^1^,^21^,^28^,^29^,^30^. The residues from G14 to R19, or from K16 to N25, of the H4 N-terminal tail are responsible for this inter-nucleosome interaction^1^,^27^.

The positively-charged lysine and arginine residues in the N-terminal tails of the core histones undergo post-translational modifications (PTMs), such as lysine acetylation and lysine/arginine methylation, in the context of chromatin^31^. These PTMs of the core histones play crucial roles in controlling the active/inactive statuses of the chromatin, and thereby regulate eukaryotic DNA-mediated metabolism, including gene expression. The PTMs of the N-terminal histone tails provide inheritable landmarks for chromatin-associated factors containing a binding domain, such as an acetyllysine-binding bromodomain or a methyllysine-binding domain^32^. In contrast, the acetylation of the H4 N-terminal tail itself markedly decreases its affinity to nucleosomal length DNA, in a cation-dependent manner^33^. Interestingly, oligonucleosome arrays containing hyper-acetylated core histones have a significantly reduced propensity to fold into higher-order chromatin structures and self-associate into tertiary structures, as compared with unacetylated arrays (reviewed in^25^). Furthermore, the single acetylation of H4-K16 disrupts the formation of the 30 nm-like fibers and the cross-fiber interactions in a reconstituted system^34^. The H4-K16Q mutation, which mimics K16ac, also leads to the structural disorder of the basic patch residues (K16-R19) of H4 in mono-nucleosomes^35^.

These observations suggest that the acetylation of the nucleosome itself regulates the decompaction of the higher-order chromatin structure, even in the absence of chromatin-associated factors. However, from the viewpoint of X-ray crystallography, it remains unclear whether the lysine acetylation of the N-terminal tails of the core histones affects the intra-nucleosomal histone•DNA interactions and/or the inter-nucleosomal histone•histone and histone•DNA interactions.

The amount of structural information about NCPs containing site-specific PTMs is quite limited. Lu *et al.* reconstituted NCPs containing aminoethylcysteine derivatives, N-dimethyl-aminoethylcysteine (Kcme2) and N-trimethyl-aminoethylcysteine (Kcme3) at H3-K79 and H4-K20, respectively, in order to mimic the methylated statuses of the lysine residues (*i.e.,* H3-K79me2 and H4-K20me3, respectively) through chemical modification of the cysteine residues, and solved their structures^36^. To our knowledge, this is the only reported crystal structure of a NCP with a PTM or its mimic. Additionally, the crystal structural analyses revealing the recognition of PTMs of core histones have been limited to the peptide level, using a modified histone peptide as a ligand, because of the difficulty in reconstituting sufficient amounts of ‘epigenetic’ NCPs with crystallization quality.

Regarding a NCP with a genuine PTM, Neumann *et al.* reconstituted the NCP containing the site-specific acetylation at H3-K56 (*i.e.,* H3-K56ac), through the genetic incorporation of acetyllysine into histone H3, and biochemically showed that the acetylation of H3-K56 does not directly affect chromatin compaction^37^. We have been developing technologies to incorporate a variety of non-natural amino acids into proteins at defined sites, through expansion of the genetic code and engineering of the translation termination system^38^,^39^,^40^,^41^. By integrating the synthetic biological technologies of cell-free protein synthesis^42^ and highly efficient amber suppression^39^, we synthesized and purified full-length histone H4 proteins with acetyllysine incorporated at multiple sites, in a site-directed manner. In this study, to understand the effects of the acetylation of histone N-terminal tails in the NCP on the nucleosomal structure, we reconstituted human NCPs with histone H4 containing a series of site-specific acetyllysine(s) within its N-terminal tail. We analyzed the biochemical characteristics of the reconstituted H4-acetylated NCPs, and solved the crystal structure of the H4-K5/K8/K12/K16-tetra-acetylated NCP at 2.4 Å resolution, for comparison with the unmodified NCP.

## Results

### Synthesis of acetyllysine-incorporated histone H4

To obtain milligram quantities of histone H4 proteins containing acetyllysine(s) at specified site(s), we took advantage of the *E. coli* cell-free protein synthesis system with the expanded genetic code^39^. Through the scheme shown in Supplementary Fig. S1a, transcription and translation were coupled *in vitro*. The template DNA contained the human histone H4 ORF, with the codon(s) of the specified residue(s) replaced with the TAG triplet(s) and a terminal TAA stop codon. This template DNA was transcribed with T7 RNA polymerase, and yielded mRNAs containing UAG triplet(s) at the specified position(s). These mRNAs were subsequently used as the templates for translation in the cell-free protein synthesis system, containing the following three materials: 1) pyrrolysine-specific tRNA (tRNA^Pyl^), 2) acetyllysine (Kac), and 3) the pyrrolysyl-tRNA synthetase (PylRS) mutant, designated as KacRS_6mt. In this system, KacRS_6mt catalyzes the aminoacylation of tRNA^Pyl^ with acetyllysine, and the resultant Kac-tRNA^Pyl^ is incorporated into the histone H4 protein specifically at the UAG site(s). Since the reacted tRNA^Pyl^ can be recycled for another aminoacylation, the amount of the translated product is expected to be much higher than those generated by other methods employing chemical or ribozyme-dependent aminoacylation.

By optimization of the cell-free protein synthesis conditions, we successfully prepared milligram quantities of histone H4 proteins containing Kac at the specified single sites of K5, K8, K12, or K16, as detected by SDS-PAGE and western blotting with a series of polyclonal antibodies recognizing the respective site-specific Kacs (Lanes 1 to 10 in Supplementary Fig. S1b). Since this cell-free system lacked release factor 1 (RF1), which recognizes the termination codons UAG and UAA, the efficiency of Kac incorporation is much higher than those in the conventional systems. Indeed, we could prepare milligram quantities of histone H4 proteins containing Kac at multiple specified sites, and even obtained the K5/K8/K12/K16-tetra-acetylated histone H4, with the nearly the same yield as the other H4 species (Lanes 11 to 18 in Supplementary Fig. S1b). Furthermore, the mass spectrometry analysis confirmed that the efficiency of the quadruple incorporation of acetyllysine was nearly 100%, while the partially-acetylated or deacetylated forms were negligibly detected (Supplementary Fig. S1c and 1d). As in the previous cases with unmodified histone H4, the synthesized H4 proteins were insoluble, as assessed by centrifugation (Supplementary Fig. S1b). Therefore, we refolded each of the precipitated H4 proteins by the method utilizing 6 M guanidine, and purified the proteins by N11-tag affinity purification and cation exchange chromatography. The typical yield of the purification to near homogeneity was approximately 0.2 mg per milliliter cell-free reaction solution, which enabled us to perform biochemical and structural analyses of the NCPs containing the acetylated H4.

### Reconstitution of Kac-incorporated NCPs

Reconstitution of the NCPs was essentially performed by the salt gradient method^43^, using the Kac-incorporated histone H4 proteins (Supplementary Fig. S1e). A native PAGE analysis of the reconstituted NCPs confirmed that the NCP containing the K5/K8/K12/K16-acetylated histone H4 (H4-tetra-acetylated NCP) was reconstituted apparently as efficiently as the unmodified NCP (Lanes 1 and 3 in Supplementary Fig. S1f). The reconstituted NCPs were then purified through precipitation, by the addition of MgCl_2_ (Lanes 2 and 4). Other NCPs containing mono- or di-acetylated H4 were also reconstituted in the same manner, for subsequent biochemical analysis.

### Thermal stability of the H4-tetra-acetylated NCP

**Figure 1:**
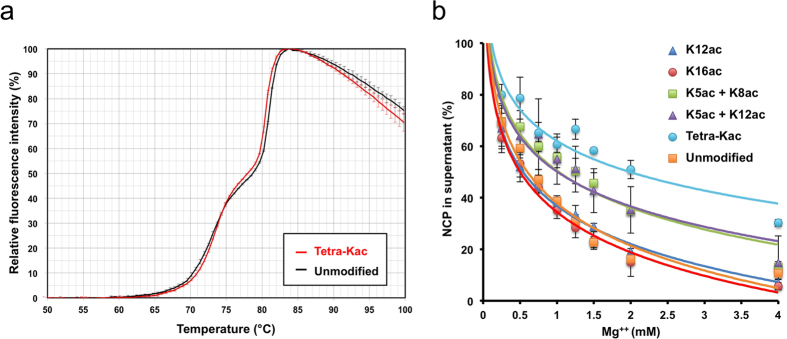
Biochemical nature of the H4-tetra-acetylated NCP. (**a**) Thermal stability analyses of the H4-tetra-acetylated and unmodified NCPs. The fluorescence intensities from 50.0 °C to 99.9 °C are plotted. Means ± SD (N = 3). (**b**) Mg^++^-dependent self-association assay of NCPs. The X- and Y-axes indicate the millimolar concentration of Mg^++^ and the percentage of soluble NCPs, respectively. Means ± SD (N = 3).

To determine the stabilities of the NCPs with or without the H4 tetra-acetylation, we performed a thermal shift assay. In this assay, the thermal stabilities were measured by monitoring the thermal denaturation profile with a fluorescent dye that binds to the hydrophobic surfaces of unfolded proteins. The eviction of histones from the NCP is detectable as an increase in the fluorescence intensity. The thermal denaturation profiles of the H4-tetra-acetylated and unmodified NCPs were quite similar to each other (Fig. 1a). Both profiles exhibited biphasic curves, indicating the stepwise evictions of the H2A-H2B dimer and the H3-H4 tetramer, which are typically detected in the thermal denaturation profiles of reconstituted NCPs^19^,^44^. The lower *T*_m_ values, reflecting the eviction of the H2A-H2B dimer, were 74.3 °C for the H4-tetra-acetylated NCP and 73.1 °C for the unmodified NCP. The higher *T*_m_ values, reflecting the eviction of the H3-H4 tetramer, were 80.8 °C for the H4-tetra-acetylated NCP and 81.4 °C for the unmodified NCP. These biochemical data indicated that the H4-tetra-acetylated NCP is as stable as the unmodified NCP *in vitro*.

### Mg^++^-dependent NCP self-association assay

Bivalent cations, such as Mg^++^, are known to facilitate the self-association of NCPs^45^,^46^,^47^. We next examined the Mg^++^-dependent self-association activities of the NCPs reconstituted with the series of acetylated H4 proteins (Fig. 1b). As compared with the unmodified NCP (orange squares), the NCPs with H4 containing one Kac at either K12 (blue triangles) or K16 (red circles) showed almost the same self-association profiles in Mg^++^-dependent manners. By contrast, the K5/K8/K12/K16-tetra-acetylated NCP showed the lowest degree of self-association among all of the NCPs tested (Fig. 1b, cyan circles). The NCPs containing two Kac residues at either K5 and K8 (green squares) or K5 and K12 (purple triangles) showed intermediate degrees of self-association between the NCPs containing non-/mono-acetylated H4 and tetra-acetylated H4. As expected from previous observations^47^, we detected a weaker tendency of self-association with a larger number of H4 N-terminal tail acetylations.

### Crystallization of the H4-tetra-acetylated NCP

Subsequently, we tried to solve the crystal structure of the Kac-incorporated NCP, to examine the influence of the H4 tail acetylation on the nucleosomal structure (Supplementary Fig. S2). The H4 protein synthesized with the initial cell-free expression construct, which we used for the aforementioned functional analyses, was non-specifically digested when its N-terminal histidine tag was removed by thrombin cleavage. Therefore, we created another cell-free expression construct, in which the thrombin recognition sequence (LVPRGS) was replaced with the TEV recognition sequence (HENLYFQ). Using this construct and TEV protease, we prepared the H4 protein tetra-acetylated at K5, K8, K12 and K16. Subsequently, we reconstituted the H4-tetra-acetylated NCP to near homogeneity for crystallization. In addition, although the crystal structures of unmodified NCPs are known, we prepared the unmodified NCP by a similar method, and crystallized it in order to compare the structures between the H4-tetra-acetylated and unmodified NCPs in detail, under controlled experimental conditions for sample preparation and crystallization. We overexpressed the unmodified H4 protein in *E. coli*, and used it for the subsequent crystal structure analysis. The sequences of the H4-tetra-acetylated and unmodified H4 proteins are the same, except that the GM and GSHM sequences, respectively, remain at their N-termini after tag removal. The SDS-PAGE profiles of the purified histone proteins used for crystallization are shown in Supplementary Fig. S2a. The faster migration of the H4-tetra-acetylated H4 protein than the unmodified H4 protein is likely to be attributable to the reduction in the number of positive charges. From single crystals of the H4-tetra-acetylated and unmodified NCPs (Supplementary Fig. S2b and c, respectively), we obtained X-ray diffraction data sets at BL32XU of SPring-8. The statistical parameters of the data collection are summarized in Table 1.

To exclude the possibility that the H4 protein became deacetylated during the incubation for crystallization, we collected the crystallization solution from the drops that yielded single crystals of the H4-tetra-acetylated NCP, and examined whether the site-specific acetylation of the H4 protein remained by western blotting. The site-specific acetylation of H4 was confirmed at all four of the sites, by immunoblotting with the residue-specific acetyllysine recognition antibodies (Supplementary Fig. S2d), demonstrating that the H4-tetra-acetylated NCP used for crystallization was not deacetylated in the crystallization process.

### Overall structure of the H4-tetra-acetylated NCP

**Figure 2:**
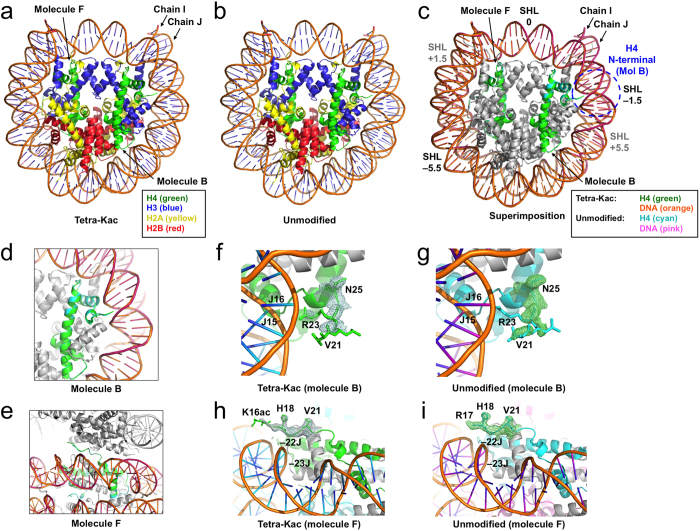
Crystal structures of the H4-tetra-acetylated NCP. (**a**) The H4-K5/K8/K12/K16-tetra-acetylated NCP. (**b**) The unmodified NCP. (**c**) Superimposition of the two NCPs. Color codes for H4 and DNA are indicated at the bottom. The possible position for the molecule-B H4 tail around SHL –1.5 is shown by a dashed blue circle. The positions of the SHLs, at –5.5, –1.5, 0, +1.5, and +5.5, are indicated. SHLs +1.5 and +5.5, which are located behind the two DNA duplexes, are indicated in gray. (**d**) Superimposition of the molecule-B H4 structures. (**e**) Superimposition of the molecule-F H4 structures (horizontal view). The symmetrically-related NCP is depicted in gray. (**f,g**) Close-up views of the molecule-B N-terminal region in the H4-tetra-acetylated (**f**) and unmodified (**g**) NCPs. Both meshes indicate the 2mFo-DFc electron density maps corresponding to residues V21–I26. Residues 1–20 are disordered. (**h,i**) Horizontal views of the molecule-F N-terminal region in the H4-tetra-acetylated (**h**) and unmodified (**i**) NCPs. The meshes correspond to residues K16ac–V21 and R17–V21, respectively. All maps are contoured at 1.5 σ.

The crystal structures of the H4-tetra-acetylated and unmodified NCPs were refined at 2.4 Å  and 2.2 Å resolutions, respectively, by molecular replacement as described in the Materials and Methods (Fig. 2). To exclude model bias, we used the auto-modeling program AutoBuild (PHENIX)^48^. Consequently, the *R*-factor/*R*_free_ decreased by several percentage points. The final refinement statistics are summarized in Table 1. We thus solved the structures of the H4-tetra-acetylated and unmodified NCPs (Fig. 2a,b, respectively). In the electron density map, both ends of the 147-bp DNA were clearly observed in the H4-tetra-acetylated NCP, as also observed for the unmodified NCP in the present and previous^49^,^50^ studies using the 147-bp DNA. The histone-fold core regions were also clearly visible in both NCPs (Fig. 2c-e). Overall, the two NCP structures were nearly identical, as demonstrated by superimposition (Fig. 2c). In the superimposition of the atomic models of the H4-tetra-acetylated NCP structure and the unmodified NCP, the global RMSD (root-mean-square deviation) values of all of the protein Cα atoms and the DNA sugar–phosphate backbone were as small as 0.22 Å and 0.27 Å, respectively. Between the H4-tetra-acetylated NCP structure and the previous Rb^+^-coordinating NCP147 structure (PDB ID: 3MGR)^50^, which exhibits the clearest electron density, the highest resolution, and the lowest RMSD values thus far, the RMSD values of all of the Cα atoms and the DNA sugar–phosphate backbone were 0.31 Å and 0.45 Å, respectively. The paths of the nucleosomal DNA superimposed well throughout the entire DNA duplex (Fig. 2c–e).

### Electron density map around the tetra-acetylated H4 tail

The structural comparison around the H4 N-terminal tail (residues 1–23) is shown in Fig. 2d–i. In the H4-tetra-acetylated NCP structure, the electron density corresponding to the residues from S1 to K20 of H4 (molecule B) is missing (Fig. 2f), which is quite consistent with the previous structural analysis^4^. The main chains from V21 to I26 and the side chains from R23 to I26 are visible in one of the H4 molecules (molecule B) of the H4-tetra-acetylated NCP (Fig. 2f). In the unmodified NCP, these features are almost completely the same: residues S1–K20 are disordered, and residues V21–I26 are visible in the molecule-B H4 (Fig. 2g). As for the other H4 molecule (molecule F), residues S1–A15 and S1–K16 are disordered and residues K16ac–I26 and R17–I26 are visible in the H4-tetra-acetylated and unmodified NCPs, respectively (Fig. 2h,i). The electron density corresponding to the acetyl group of K16 was weak in molecule F of the H4-tetra-acetylated NCP (Fig. 2h). Residues V21–I26 of molecule B superimposed extremely well between the two NCPs (Fig. 2d), while molecule B does not contribute to the interaction with the acidic patch of the neighboring NCP. Residues R17–I26 of molecule F also superimposed well between the two NCPs (Fig. 2e), while molecule F interacts with the acidic patch of the neighboring NCP. In particular, the position of R23 in molecule F and its hydrogen bonds to E56 of H2A and E110 of H2B, in the acidic patch of the neighboring NCP, are conserved between the H4-tetra-acetylated and unmodified NCPs.

### *B*-factors of the nucleosomal DNA

**Figure 3:**
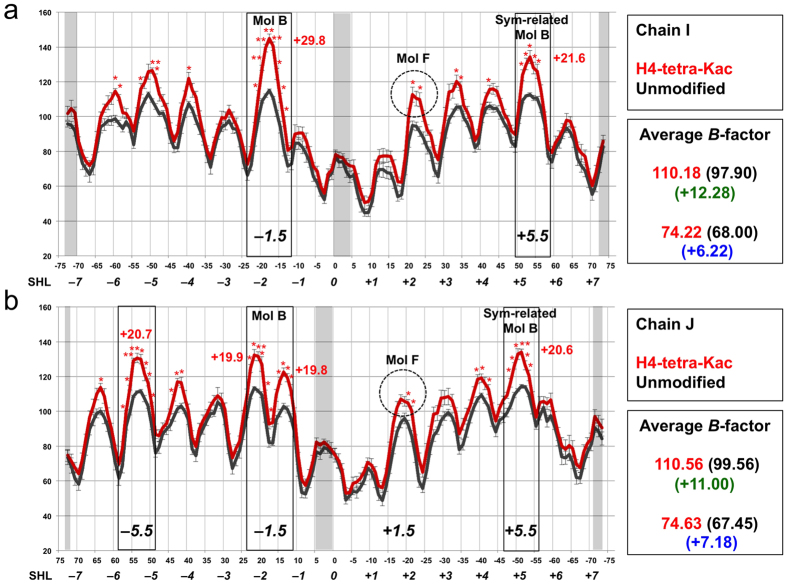
*B*-factors of the H4-tetra-acetylated NCP DNA. (**a**) *B*-factor plot of chain I. (**b**) *B*-factor plot of chain J. In both plots, the X-axis indicates the position of the DNA nucleotide and the Y-axis indicates the *B*-factor (Å^2^). Superhelical locations (SHLs) are shown at the bottom of the X-axis. In both DNA chains, the average of the *B*-factors of all of the modeled atoms of the nucleotide, from three independently refined structures of the H4-tetra-acetylated NCPs (red) and unmodified NCPs (black), are respectively presented as means ± SD at each nucleotide. Asterisks: *P < 0.05 and **P < 0.01 in two-tailed Student’s *t*-test. The regions near the dyad and both DNA termini are colored gray where the *B*-factor differences around the tops of the cyclical pattern are not significant. The solid rectangle indicates the position where the visible N-terminal residue of the molecule-B H4 (SHL –1.5) or that of the symmetrically-related molecule-B H4 (SHL +5.5) is located within 20 Å. The position where the visible N-terminal residue of the molecule-F H4 is located nearby is indicated by the dashed circle (SHL +2). Average scores of the *B*-factors of each DNA chain are shown in the boxes on the right, in the following order (from top to bottom): cyclical top average scores (the H4-tetra-acetylated NCP in red and the unmodified NCP in black); the top average difference in the *B*-factor scores in green; the cyclical bottom average scores (the H4-tetra-acetylated NCP in red and the unmodified NCP in black); and the bottom average difference in the *B*-factor scores in blue.

Next, we compared the *B*-factors of the nucleosomal DNA between the H4-tetra-acetylated and unmodified NCPs. The averaged *B*-factors from the three independently refined structures of the H4-tetra-acetylated and unmodified NCPs were plotted along the nucleotide sequence (Fig. 3). In general, the *B*-factors of the outward DNA superhelix in almost every superhelical location (SHL) are large, whereas those of the positions facing the histone octamer are small in the NCPs^1^. In the present study, the H4-tetra-acetylated NCP also shows a similar cyclical pattern with the tops and bottoms of the *B*-factors. Furthermore, the *B*-factor differences around the tops of the cyclical pattern, or the outward DNA superhelices, between the H4-tetra-acetylated and unmodified NCPs are statistically significant, except for the regions near the dyad and both DNA termini (Fig. 3a,b). The averages of the *B*-factors of the bottoms, or the histone-facing nucleotides, are 74.2 Å^2^ in chain I (Fig. 3a) and 74.6 Å^2^ in chain J (Fig. 3b) in the H4-tetra-acetylated NCP, and slightly larger than those in the unmodified NCP (68.0 Å^2^ and 67.5 Å^2^, respectively). In contrast, the averages of the *B*-factors of the tops in the cyclical pattern are 110.2 Å^2^ (chain I; Fig. 3a) and 110.6 Å^2^ (chain J; Fig. 3b) in the H4-tetra-acetylated NCP, and also slightly larger than those in the unmodified NCP (97.9 Å^2^ and 99.6 Å^2^, respectively). However, the *B*-factor differences at the two tops around SHLs –1.5 and +5.5 in chain I, and the three tops around SHLs –5.5, –1.5, and +5 in chain J between the H4-tetra-acetylated and unmodified NCPs are statistically significant, and are marked with rectangles and asterisks (*P < 0.05 and **P < 0.01) in Fig. 3a,b.

The DNA positions in which the backbones are located within 20 Å from either of the visible N-terminal residues of the H4 tails from molecules B and F, and the symmetrically-related molecule B, are indicated in Fig. 3. In the H4-tetra-acetylated NCP, the outward DNA around SHL –1.5 exhibits peaks with statistically significant *B*-factor differences (19.8, 19.9, and 29.8 Å^2^), and is located within 20 Å from the visible N-terminal residue of the intra-nucleosomal molecule-B H4. The DNA around SHL +5.5 also exhibits peaks with statistically significant *B*-factor differences (20.6 and 21.6 Å^2^), and is near the visible N-terminal residue of the inter-nucleosomal molecule-B H4 (Fig. 3a,b). In addition, a peak with a significant *B*-factor difference (20.7 Å^2^) is observed around SHL –5.5 in chain J (Fig. 3b). This region of DNA does not interact with the H4 molecules, but is located near the visible N-terminal residue of the molecule-D H2B and SHL –2 of the symmetrically-related chain J, as described in the Discussion.

**Figure 4:**
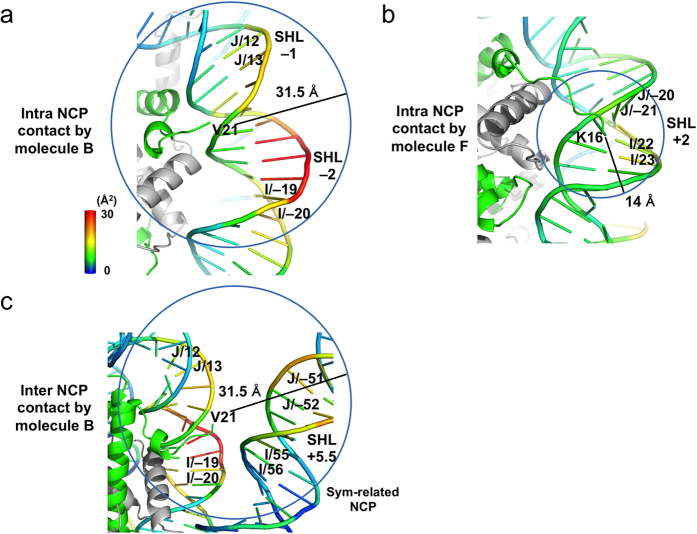
Representation of *B*-factor-increased nucleosomal DNA regions by the H4 tetra-acetylation. (**a**) Intra-nucleosomal contacts by the molecule-B H4. The DNA chains are color-coded by the difference in the *B*-factors between the H4-tetra-acetylated and unmodified NCPs. The color bar represents the *B*-factor difference from 0 Å^2^ (blue) to 30 Å^2^ (red). (**b**) Intra-nucleosomal contacts by the molecule-F H4. (**c**) Inter-nucleosomal contacts by the molecule-B H4.

In contrast, the outward DNA around SHL +2 is near the visible N-terminal residue of the intra-nucleosomal molecule-F H4, but its *B*-factor differences are comparatively modest (dashed circles in Fig. 3a,b). Close-up views of the nucleosomal DNAs near the H4 tail of molecule B or F are shown in Fig. 4a–c. The positions of the DNA backbone with large *B*-factor differences around 30 Å^2^ are color-coded in red. As residues 1–20 and 1–15 of molecules B and F, respectively, are disordered in the crystals, we roughly estimated the distances from the visible N-terminal residues, V21 of molecule B and K16ac of molecule F, to one of the acetyllysine-incorporated residues, K12ac, using the average inter-residue distance of approximately 3.5 Å in a typical β strand, as 31.5 Å (9 residues) and 14 Å (4 residues), respectively. These distances are indicated as the radii of the circles. From this representation, we found that although the electron densities corresponding to the N-terminal 20 residues are disordered in the molecule-B H4, the H4-tetra-acetylation of the molecule-B H4 seems to locally affect the *B*-factors of both the intra-nucleosomal DNA (Fig. 4a) and inter-nucleosomal DNA (Fig. 4c). However, the H4-tetra-acetylation of the molecule-F H4 affected the *B*-factors of the nearby intra-nucleosomal DNA to a lower extent (Fig. 4b; see also dashed circles in Fig. 3a,b). Finally, we averaged the *B*-factors of all nucleotides, except the significantly affected ones, for each of the DNA chains, and confirmed that there was no statistically significant effect of the H4-tetra-acetylation.

### *B*-factors of the histones

We next investigated the *B*-factor differences of the nucleosomal histones between the two NCPs. Among the three independently refined structures, the *B*-factor of every residue was averaged in each of the H4-tetra-acetylated and unmodified NCPs. The average *B*-factors of each of the histones (molecules A–H) were plotted along its amino acid sequence (Supplementary Fig. S3a-h). Overall, we found that almost all of the residues of histones H3, H2A, and H2B, as well as those of H4, had slightly higher *B*-factors in the H4-tetra-acetylated NCP than in the unmodified one: the total average *B*-factors were minimally 4.23 Å^2^ higher (H3, molecule E; Supplementary Fig. S3b) and maximally 5.61 Å^2^ higher (H4, molecule B; Supplementary Fig. S3c). These differences in the *B*-factors of the histones are approximately 3- to 4-fold smaller than the significant *B*-factor differences observed in the outward DNAs around SHLs –1.5 and +5.5 (Fig. 3).

The core region of each histone scarcely showed a statistically significant *B*-factor difference between the two NCPs. The histone residues with the statistically significant *B*-factor differences are located at or near the interfaces of the inter- or intra-nucleosomal interactions (Table 2). For example, F78 of the molecule-A H3 is located near Q76, which forms an inter-nucleosomal interaction with the chain-J dC–62. In contrast, several N-terminal tail residues of the molecule-B H4 (Supplementary Fig. S3c) and the molecule-D H2B (Supplementary Fig. S3g) exhibited significantly larger *B*-factor differences. In the molecule-B H4, which does not interact with the neighboring NCP, a stretch of N-terminal residues from V21 to N25 showed statistically significant *B*-factor differences (P < 0.05) (Supplementary Fig. S3c; Table 2). In contrast, the molecule-F H4 interacts with the neighboring NCP, mainly through the interaction of R23 with the acidic patch region of the molecule-C H2A/the molecule-D H2B heterodimer^1^, which critically contributes to the formation of the higher-order chromatin structure^14^. Between the H4-tetra-acetylated and unmodified NCPs in this study, R23 does not exhibit a statistically significant *B*-factor difference, and only H18 (P = 0.045) interacting with H2A-D90 showed significantly larger *B*-factor differences (Supplementary Fig. S3d; Table 2). In the other histones than H4, the N-terminal tail residues from S32 to E35 of the molecule-D H2B showed significantly higher *B*-factor differences between the two NCPs (Supplementary Fig. S3g; Table 2). This region of H2B is located near SHL –5 and SHL +3 within the NCP. In this stretch, the side chain of the molecule-D S32 (P = 0.019) hydrogen bonds with the backbone phosphate group of the chain-I dG30 around SHL +3. Around SHL –5, the main chain of the molecule-D K34 (P = 0.031) hydrogen bonds with the backbone phosphate group of the chain-J dT50.

**Figure 5:**
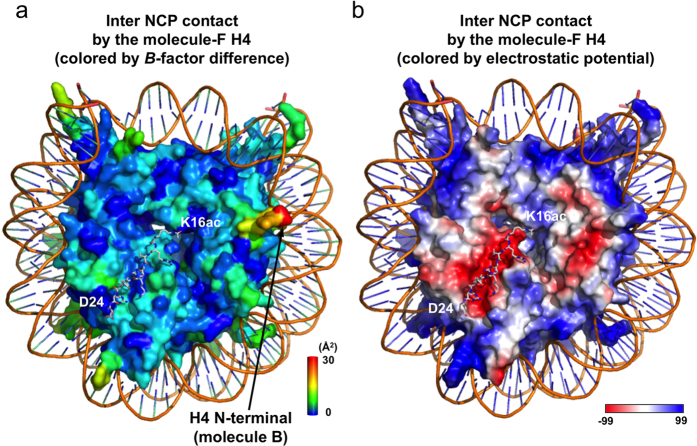
Nucleosomal histone regions with increased *B*-factors by the H4 tetra-acetylation. (**a**) Inter-nucleosomal contact by the molecule-F H4. The color bar represents the *B*-factor difference between the two NCPs from 0 Å^2^ (blue) to 30 Å^2^ (red). The residues K16ac–D24 of the symmetrically-related molecule-F H4 are depicted by gray sticks. (**b**) Inter-nucleosomal contact by the molecule-F H4. The histone surface is color-coded by the electrostatic potential. Red and blue colors indicate acidic and basic parts of the histone octamer surface, respectively. Note that the H4 tail-interactive acidic patch surface does not exhibit a large *B*-factor difference.

We also examined the *B*-factor differences of the acidic patch residues in histones H2A and H2B. The acidic patch of molecules C and D (H2A/H2B) associates with the H4 tail of molecule F of the symmetrically-related NCP, while that of molecules G and H (H2A/H2B) does not^1^. The acidic patch-composing residues of molecules C, D, G, and H lacked statistically significant *B*-factor differences. Near the acidic patch, V48 of the molecule-D H2B contributes to the inter-nucleosomal interaction with V21 of the molecule-F H4, with a significant *B*-factor difference (P = 0.031), whereas the *B*-factor of V21 of the molecule-F H4 is not significantly affected (P > 0.05) (Supplementary Fig. S3e-h; Table 2). To visualize the effect of the H4 tail tetra-acetylation on the histone core region, we mapped the *B*-factor differences of all four histones on the crystal structure of the H4-tetra-acetylated NCP (Fig. 5a). The observation that the acidic patch surface (Fig. 5b) lacks a significantly larger *B*-factor difference (Fig. 5a; see also Supplementary Fig. S3e-h) supports the conclusion that the inter-nucleosomal histone•histone interactions are not significantly affected by the H4 tail tetra-acetylation. Finally, we averaged the *B*-factors of all amino acid residues, except the significantly affected residues, for each of the histone molecules, and confirmed that there was no statistically significant effect of the H4-tetra-acetylation.

### Binding activities of the acetylated NCPs to the TAF1 double bromodomain

**Figure 6:**
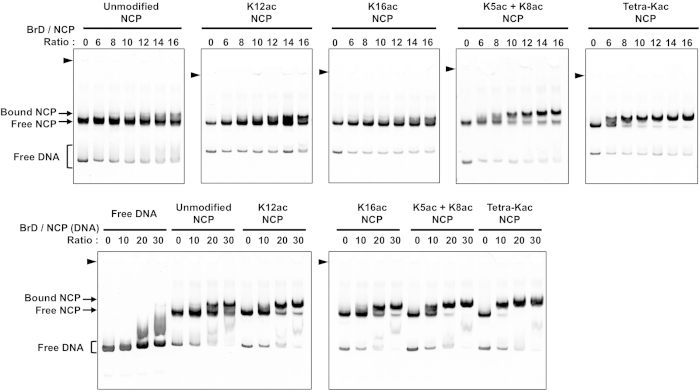
Electrophoresis mobility shift assay of the binding between H4-acetylated NCPs and the TAF1 double bromodomain (TAF1-BrD). The indicated ratios of the TAF1-BrD were mixed with the respective NCPs. The bands corresponding to the TAF1-BrD-bound NCP, the TAF1-BrD-unbound NCP, and the free DNA are respectively indicated by the arrows and brackets on the left. The positions of the wells are indicated by arrowheads.

Lysine acetylation of the histone tail reportedly enhances the binding with a bromodomain, as assessed by biochemical analyses performed mostly with acetylated peptides as binding substrates, but very rarely with modified nucleosomes^37^,^51^. To validate the utility of the Kac-incorporated NCPs, we examined the stoichiometric association between the prepared Kac-incorporated NCPs and the double bromodomain of TAF1 (TAF1-BrD), which reportedly interacts with acetylated H4^52^, by a native PAGE mobility shift assay. When the unmodified NCP was used as the substrate, partial formation of the NCP•TAF1-BrD complex was detected at a high molar ratio of TAF1-BrD (Fig. 6). When the single Kac-incorporated NCPs at K12 and K16 were used, the band shifts were also modest. When the number of acetylation sites was increased to 2 (both K5 and K8) or 4 (K5/K8/K12/K16), the binding affinity of the TAF1-BrD for the acetylated NCP increased (Fig. 6). The dissociation constants estimated from triplicate electrophoresis mobility shift assays were: 19.3 ± 0.3 μM for the unmodified NCP, 12.3 ± 1.9 μM for the K12-acetylated NCP, 13.3 ± 1.6 μM for the K16-acetylated NCP, 6.1 ± 0.4 μM for the K5/K8-di-acetylated NCP, and 3.9 ± 0.2 μM for the K5/K8/K12/K16-tetra-acetylated NCP. These results indicate that the affinity of the TAF1-BrD for the NCP is enhanced by increased numbers of H4 acetylation sites, as expected. However, the gradient of the affinity is rather modest, as compared with the analysis using acetylated tail peptides^52^. We also detected the weak affinity of the TAF1-BrD to free nucleosomal DNA at a high molar ratio (Fig. 6, bottom), which may be a reason for the modest affinity gradient for the NCP (see Discussion).

## Discussion

We solved the crystal structure of an NCP containing genuine PTMs. Through the utilization of genetic code-expansion technologies to synthesize acetyllysine-incorporated proteins, and the subsequent reconstitution of human NCPs with the ‘genetically’ acetylated H4 proteins (Supplementary Fig. S1), we assessed how the acetylation of the H4 N-terminal tail influences its intra- and inter-nucleosomal interactions, on the basis of the crystal structures at 2.2–2.4 Å resolutions. The structure of the NCP with the K5/K8/K12/K16-tetra-acetylated H4 is fundamentally the same as that of the unmodified NCP. Specifically, the H4 tetra-acetylation does not affect the structure of the histone octamer, the conformations of the visible parts of the histone tail regions, including the two H4 N-terminal regions (*i.e.,* V21–I26 in molecule B and R17–I26 in molecule F), or the superhelical structures of the DNA up to both ends (Fig. 2). The H4 tetra-acetylation significantly increases the *B*-factors of the outward DNAs in 7–8 SHLs, particularly around SHLs –1.5 and +5.5 (Figs 3 and 4). By contrast, the *B*-factors of the inward DNAs and the regions near the dyad and both DNA termini are scarcely affected, and the *B*-factors of the histones, including the acidic patch residues, are negligibly affected (Fig. 5; Supplementary Fig. S3). We also performed biochemical analyses of the H4 tetra-acetylated and unmodified NCPs. The thermal stability of the NCP was not affected by the H4 tetra-acetylation (Fig. 1a). In contrast, the NCPs with different patterns of H4 acetylation showed distinct profiles of self-association (Fig. 1b) and bromodomain-binding activities (Fig. 6).

First, we addressed the effect of the H4 tetra-acetylation at K5/K8/K12/K16 on chromatin compaction. Analyses of the H4-K16-acetylated nucleosome prepared by native chemical ligation demonstrated that the acetylation of H4-K16 in the nucleosome array inhibits the formation of 30 nm fibers and cross-fiber interactions^34^, indicating the critical role of the H4-K16 acetylation in chromatin decompaction. Moreover, biochemical analyses of various acetylated NCPs, prepared by native chemical ligation, revealed that cation-induced self-association was reduced by the H4-K5/K8/K12/K16-tetra-acetylation^47^. Our genetically-prepared H4-K5/K8/K12/K16-tetra-acetylated NCP also exhibited reduced cation-induced self-association (Fig. 1b). The single acetylation of H4-K16 showed a smaller decrease in self-association in the present study, as compared to previous results^47^. Our observations are consistent with the idea that the cation-induced self-association is mainly governed by a general electrostatic mechanism^47^,^53^.

On the basis of the *B*-factor differences, the present crystallographic study clarified that the H4 N-terminal tail interacts with the intra- and inter-nucleosomal DNAs, and the H4 tetra-acetylation decreases these H4 tail•DNA interactions, although the individual structure of the H4-tetra-acetylated NCP is nearly the same as that of the unmodified NCP (Fig. 2). In detail, the tetra-acetylation of the H4 N-terminal tail significantly increases the *B*-factors of the outward DNAs (Figs 3 and 4). In the unmodified NCP, the *B*-factor tops and bottoms, corresponding to the outward and inward DNAs, respectively, periodically appear along the DNA^1^. The *B*-factors of the outward DNAs in the H4-tetra-acetylated NCP are higher than those in the unmodified NCP. The most prominent *B*-factor increases are around SHLs –1.5 and +5.5, which are both located near the putative positions of the N-terminal tails of the molecule-B H4 (SHL –1.5) or the adjacent symmetrically-related molecule-B H4 (SHL +5.5) (Fig. 3). The significant *B*-factor differences around SHLs –1.5 and +5.5 suggest that the H4 tetra-acetylation causes more fluctuation of the atomic positions around the two SHLs. Thus, it is unambiguously concluded that intra- and inter-nucleosomal interactions exist between the DNA and residues 1–16 or 1–20 of the H4 tail, which are disordered in the crystal structures, and that the tetra-acetylation of the tail impairs these interactions (Fig. 4).

In the chain-J DNA strand, a significant *B*-factor increase was also detected near SHL –5.5 (*i.e.,* from dT50 to dT57), with which the N-terminal tail of the molecule-D H2B partially interacts. The inter-nucleosomal histone•DNA interaction *via* the N-terminal tail of the molecule-D H2B mainly occurs at three chain-J nucleotides around this region: dA51 (P = 0.017) interacting with H2B-R31, dT50 (P = 0.025) interacting with H2B-K34, and dG49 (P > 0.05) interacting with H2B-S36. The other nucleotides with significant *B*-factor increases around SHL –5.5, especially those from dG52 to dT57, are not involved in the inter-nucleosomal interactions, but they are close to SHL –2 of the symmetrically-related chain J (this study and reference^1^). Therefore, the effects of the H4 tetra-acetylation on the other outward DNAs, such as that around SHL –2 of the symmetrically-related NCP, may affect the properties of SHL –5.5. It should be noted here that the DNA structures around SHL ±5 and SHL ±2 can exhibit extreme kinks^54^.

In the H4 N-terminal region, residues K16–N25 are considered to form inter-nucleosomal interactions with the acidic side chains of E56, E61, E64, D90, E91, and E92 of H2A^1^. We clarified that the H4-K5/K8/K12/K16-tetra-acetylation does not affect the histone•histone interactions between the N-terminal tail and the acidic patch (Figs 2e and 5; see also Supplementary Fig. S3). H4-K16 was also predicted to hydrogen bond with the main-chain carbonyl groups of R96, L97, and L99 of H2B^53^. Our structural study revealed that the H4 tetra-acetylation does not affect the structures and *B*-factors of the H2B residues R96, L97, and L99. However, we cannot discuss the direct interaction between H4-K16 and these H2B residues, because the K^+^ ion in the crystal may inhibit this interaction^53^. Interestingly, the inter-nucleosomal histone•histone interaction between the N-terminal H4 tail (molecule F) and the H2A/H2B acidic patch (molecules C and D) is not affected by the H4 tetra-acetylation with respect to the *B*-factor differences, except for the interaction between H4-H18 (P = 0.045) and H2A-D90. Therefore, the present crystal structure analysis suggests, on the basis of the *B*-factor differences, that the simultaneous acetylations at K5, K8, K12, and K16 in the H4 N-terminal tail do not affect the inter-nucleosomal interactions of the H4 tail region with either H2A or H2B, which constitute the acidic patch together. The inter-nucleosomal H4 tail•acidic patch interaction is only negligibly affected by the H4 tetra-acetylation, while the statistically affected histone core residues are mostly located at or near the inter-nucleosomal interfaces (Table 2).

Taken together, the H4 N-terminal tail is involved in at least two different modes of interactions: 1) the structurally disordered ‘flexible tail’ region, S1–K20 (molecule B) or S1–K16 (molecule F), interacts with the nucleosomal DNA; and 2) the structurally ordered ‘solid tail’ region, R17–R23 (molecule F) or V21–R23 (molecule B), interacts with the acidic patch of the nearby NCP. The ‘flexible tail’•DNA interactions are likely to contribute to nucleosome compaction, for the following reasons. First, the positively-charged lysine residues in the H4 ‘flexible tail’ region interact with the negative charges of the DNA, which may reduce the electrostatic repulsions between nucleosomes. Furthermore, the inter-nucleosomal H4 tail•DNA interaction may stabilize the higher-order chromatin structures. The H4 tetra-acetylation deprives the tail of positive charges, and therefore causes inter-nucleosomal decompaction (Fig. 1b and reference^47^). Thus, the H4 acetylation leads to structural changes in the higher-order chromatin, eventually resulting in transcriptional activation. In contrast, the H4 tail•DNA interaction may not contribute to DNA folding around the histone octamer, as the H4 tetra-acetylation does not affect the thermal stability of the NCP (Fig. 1a).

In contrast to the flexible tail region, the ‘solid tail’ region R17–R23/V21–R23 is likely to contribute to the inter-nucleosomal histone•histone interactions by binding to the acidic patch of the nearby NCP, mainly through R23^14^, regardless of the acetylation state. Interestingly, this ‘solid tail’ region around V21 may also contribute to acetylation-independent, inter-nucleosomal histone•DNA interactions^29^, when it is exposed to the solvent. The relative inter-nucleosomal arrangements of the H4 solid tail and the neighboring acidic patch are different between the crystal structures, including the present ones, and the inter-tetranucleosomal cryo-EM structures of reconstituted 30 nm fiber-like particles^14^, and there may be additional arrangements in the nucleus. Nevertheless, the inter-nucleosomal histone•DNA interactions, and their alterations by the H4 tetra-acetylation, suggested by present crystallographic analysis, may play a regulatory role in the maintenance of the higher-order chromatin structure in the nucleus.

In addition to the abovementioned structural features of the H4-tetra-acetylated NCP, we also examined the preference of the acetyllysine-binding double bromodomain of TAF1 (TAF1-BrD) to the series of H4-acetylated NCPs (Fig. 6). The previous isothermal titration calorimetric analysis utilizing acetylated H4 peptides demonstrated that the TAF1-BrD binds to histone H4 tail peptides with the following dissociation constants: unmodified H4 tail, larger than 250 μM (19.3 ± 0.3 μM in the present EMSA); K16-acetylated H4 tail, 39 ± 7 μM (13.3 ± 1.6 μM); and K5/K8/K12/K16-tetra-acetylated H4 tail, 5.3 ± 0.2 μM (3.9 ± 0.2 μM)^52^. The present EMSA analysis utilizing the acetylated NCPs similarly confirmed that increased acetylations of the H4 N-terminal tail facilitate the interaction with the TAF1-BrD. However, in the cases of NCPs, the affinity is modestly strengthened by the increased acetylation, and even the unmodified NCP interacted with the TAF1-BrD. This discrepancy may be partially caused by the different analysis methods. Another possible interpretation is that the NCP can function as a binding scaffold for the TAF1-BrD even in the absence of the H4 tail acetylation, possibly through a region other than the H4 N-terminal tail. Indeed, the TAF1-BrD had weak affinity to free nucleosomal DNA (Fig. 6). Hence, the interaction between the NCP and the TAF1-BrD may be multivalent, and the basal level of interaction between the unmodified NCP and the TAF1-BrD is strengthened by the H4 tail acetylation in the NCP.

The confirmation of the incorporation of site-directed acetylation (Supplementary Fig. S1b), the successful reconstitution of NCPs (Supplementary Figs. S1d and S2), and the functional differences biochemically detected among the tested NCPs (Figs 1b and 6) ensure that the current methodology for the preparation of genuinely acetylated NCPs has reached the biochemical analysis grade. Moreover, we have demonstrated that the crystal structure of the NCP prepared with the quadruple acetyllysine-incorporated H4 is essentially the same as that of the unmodified NCP (Fig. 2). This result not only indicates that the H4 acetylation does not affect the structure of the individual NCP, but also suggests that the structure of the NCP reconstituted using pre-acetylated histone H4 is likely to reflect the intact structure of the post-translationally acetylated NCP. The evidence that the H4-tetra-acetylated NCP had essentially the same tertiary structure and stability as the unmodified NCP, without any significant changes in the histone•DNA interactions as observed in NCPs lacking the N-terminal tail^19^, ensures the integrity of the reconstituted, site-specifically acetylated NCPs at the crystallization level. Hence, the present methodology for reconstituting acetyllysine-incorporated NCPs presumably reflects the intact nature of post-translationally acetylated NCPs, and will be pivotal for future structural analyses of precisely acetylated nucleosomes.

## Methods

### Preparation of site-specifically acetyllysine-incorporated histone H4 proteins

The acetyllysine (Kac)-incorporated histone H4 proteins were synthesized *in vitro,* essentially as previously described^39^. Briefly, the human histone H4 cDNA, containing from one to four TAG codons at designated site(s) for Kac incorporation and a TAA codon for translation termination, was subcloned into pCR2.1 (Invitrogen). The histone H4 protein was designed to have an N-terminal histidine-rich affinity tag (N11-tag) followed by a thrombin- or TEV-protease recognition sequence, MKDHLIHNHHKHEHAHALVPRGSHM (N11-thrombin) or MKDHLIHNHHKHEHAHAHENLYFQGM (N11-TEV), respectively. The H4 proteins were synthesized by the cell-free protein synthesis method^42^, using the engineered pyrrolysyl aminoacyl tRNA synthetase (PylRS) containing the six point mutations of N203T, L301I, L305I, Y306F, L309A, and C348F (KacRS_6mt), the UAG-recognizing tRNA^Pyl^, and the S30 extract prepared from *prfA*-eliminated “RFzero” *Escherichia coli* BL21 cells^39^. Cell-free synthesis in the dialysis mode was performed by an overnight incubation at 30 °C, using 30 cm^2^ of dialysis membrane per ml reaction solution. The synthesized H4 proteins were insoluble. The precipitates were solubilized in 50 mM Tris-HCl buffer (pH 8.0) containing 6 M guanidine-HCl, 500 mM NaCl, and 15 mM imidazole, loaded onto a HisTrap 1 ml HP column (GE Healthcare), and eluted with an imidazole gradient from 15 mM to 500 mM. The H4 proteins were subjected to ion-exchange chromatography on a TSKgel SP-5PW column (Tosoh Corporation), and then dialyzed against distilled water containing 5 mM 2-mercaptoethanol. The N11-thrombin-tagged H4 proteins, with or without Kac, were lyophilized and used for biochemical analyses. The N11-TEV-tagged, K5/K8/K12/K16-tetra-acetylated H4 protein was separated from the N11-tag by TEV protease, purified, lyophilized and used for crystallization. Histone proteins were also expressed in *E. coli*. The unmodified histone H4 protein used for crystallization was recombinantly expressed in *E. coli* JM109 (DE3), and prepared as previously described^55^. The unmodified histones H2A type 1-B/E, H2B type 1-J, and H3.1, hereafter referred to simply as H2A, H2B, and H3, respectively, for both the crystallographic and biochemical analyses were recombinantly expressed in *E. coli* BL21 (DE3), and prepared as previously described^55^.

### Reconstitution of the NCPs

The H4-acetylated and unmodified NCPs were reconstituted essentially as described^43^,^56^, using the acetyllysine-incorporated and unmodified H4 proteins, the unmodified histones H2A, H2B, and H3, and a 147-bp palindromic DNA fragment of the human α-satellite DNA region that was constructed from the plasmid NCP147, a kind gift from Dr. T. J. Richmond^49^. To purify the NCPs from the free nucleosomal DNA, MgCl_2_ was added to the reconstituted NCP solutions at a final concentration of 12 mM. The solutions were incubated for 15 min at room temperature, and the NCPs were precipitated by centrifugation at 17,500 *g* for 10 min at 4 °C. After centrifugation, the supernatant containing the free nucleosomal DNA was removed, and the precipitated NCPs were used for biochemical and crystallographic analyses.

### Thermal stability assay

The thermal stabilities of the H4-tetra-acetylated and unmodified NCPs were measured essentially as previously described^44^. Briefly, 20 pmol NCPs in 20 mM Tris-HCl buffer (pH 7.5), containing 1 mM EDTA and 1 mM dithiothreitol, were reacted with Protein Thermal Shift Dye (Life Technologies) in a 20 μl reaction volume. Fluorescence intensities were monitored at every 4 sec with a temperature change of 0.9 °C/min from 25.0 °C to 99.9 °C, using a QuantStudio 6 PCR system (Life Technologies). Three independent data sets were obtained and averaged. The data from the H4-tetra-acetylated and unmodified NCPs were normalized by setting the fluorescence intensities at 50 °C to 0%, and the maximal fluorescence intensities, which were both detected at 83.8 °C, to 100%.

### Mg^++^-dependent NCP self-association assay

The self-associations of the H4-acetylated and unmodified NCPs were measured essentially as previously described^26^,^27^. Briefly, 2.5 μM of NCPs, in 10 mM Tris-HCl buffer (pH 7.5) containing 1 mM EDTA, were mixed with an equal amount of 10 mM Tris-HCl buffer (pH 7.5) containing twice the final concentration of MgCl_2_, incubated for 15 min, and centrifuged at 16,300  *g* for 15 min at 24 °C. The absorbance at 260 nm of the supernatant was measured. The net MgCl_2_ concentration was calculated by subtracting 0.5 mM from the final concentration of MgCl_2_. Three independent data sets were obtained and averaged. The percentages of the remaining NCPs in the supernatants were plotted against the net MgCl_2_ concentrations. The absorbance at 260 nm of the supernatant containing 0 mM MgCl_2_ was set to 100%.

### Crystallization of the NCPs

The NCPs were incubated at 42 °C for 2 hrs before crystallization. Crystals were grown at 20 °C, using drops containing 4–6 mg/ml NCP, in 20 mM potassium cacodylate buffer (pH 6.0) containing 110–165 mM manganese(II) chloride and 65–95 mM potassium chloride. The crystals were transferred to 20 mM potassium cacodylate buffer (pH 6.0), containing 37 mM manganese(II) chloride, 40 mM potassium chloride, 2% trehalose, and 24% 2-methyl-2,4-pentanediol^1^, and flashed cooled with liquid nitrogen.

### Data collection and structure determination

Diffraction data were collected at 100 K at the BL32XU beam line of SPring-8 (Hyogo, Japan). The diffraction images were processed with XDS^57^ and HKL2000^58^. The structures were solved by molecular replacement (MR) with PHASER, using the structural coordinates of the 147-bp DNA in NCP147 (PDB ID: 1KX5)^49^ and the histone octamer in the human NCP (PDB ID: 2CV5)^4^ as the search models. Structural refinement was accomplished with the PHENIX suite^48^, and manual model building was performed with Coot^59^. PyMol (The PyMOL Molecular Graphics System) was used to render the structural figures and for general manipulations. The final refinement statistics are summarized in Table 1.

### Electrophoresis mobility shift assay

To analyze the binding between NCPs and the TAF1 double bromodomain (TAF1-BrD), 2.5 pmol (approximately 500 ng) of NCPs containing the series of acetylated and unmodified histone H4 proteins were mixed with the TAF1-BrD in 20 mM Tris-HCl buffer (pH 7.5), containing 40 mM NaCl, 0.75 mM EDTA, and 1 mM DTT. The samples were incubated for 1 hr at 25 °C. The TAF1-bound and -free NCPs were analyzed by native PAGE, using a gel containing 5% acrylamide (acrylamide: bisacrylamide = 29:1) in 0.2× TBE buffer, and subsequent staining with ethidium bromide or SYBR Gold nucleic acid gel stain (Life Technologies). Densitometric quantification of the gel images was performed with an LAS 4000 imager (GE Healthcare).

## Additional Information

**Accession codes**: The structural coordinates have been deposited in the Protein Data Bank, under the accession codes 5AV5, 5AVB, and 5AVC (the H4-tetra-acetylated NCPs); and 5AV6, 5AV8, and 5AV9 (the unmodified NCPs), respectively.

**How to cite this article**: Wakamori, M. *et al.* Intra- and inter-nucleosomal interactions of the histone H4 tail revealed with a human nucleosome core particle with genetically-incorporated H4 tetra-acetylation. *Sci. Rep.* **5**, 17204; doi: 10.1038/srep17204 (2015).

## Supplementary Material

Supplementary Information

## Figures and Tables

**Table 1:** Data collection and refinement statistics.

	Tetra-ac NCP No. 1	Tetra-ac NCP No. 2	Tetra-ac NCP No. 3	Unmodified NCP No. 1	Unmodified NCP No. 2	Unmodified NCP No. 3
Wavelength (Å)	1.00	1.00	1.00	1.00	1.00	1.00
Resolution range (Å)	19.98–2.40 (2.49–2.40)^§^	20.00–2.40 (2.49–2.40)	19.95–2.40 (2.49–2.40)	19.92–2.20 (2.28–2.20)	20.00–2.20 (2.28–2.20)	19.99–2.20 (2.28–2.20)
Space group	*P*2_1_2_1_2_1_	*P*2_1_2_1_2_1_	*P*2_1_2_1_2_1_	*P*2_1_2_1_2_1_	*P*2_1_2_1_2_1_	*P*2_1_2_1_2_1_
Unit cell dimensions (*a, b, c*) (Å)	106.23, 110.03, 181.88	105.95, 110.09, 181.61	106.52, 110.12, 182.55	106.39, 110.04, 182.30	106.19, 109.96, 181.89	106.26, 109.85, 182.13
Angles (*α, β, γ*) (^o^)	90, 90, 90	90, 90, 90	90, 90, 90	90, 90, 90	90, 90, 90	90, 90, 90
Total reflections	489880 (40026)	475880 (37561)	513615 (37941)	783773 (70564)	773431 (68741)	782179 (73316)
Unique reflections	83706 (8086)	83428 (8063)	84098 (7279)	108435 (10640)	107079 (10388)	108421 (10729)
Multiplicity	5.9 (5.0)	5.7 (4.7)	6.2 (5.2)	7.2 (6.6)	7.2 (6.6)	7.2 (6.8)
Completeness (%)	99.93 (99.38)	99.95 (99.66)	99.70 (99.83)	99.62 (98.87)	98.85 (97.01)	99.99 (99.98)
Mean *I*/*σ*(*I*)	8.17 (1.96)	6.85 (1.70)	8.90 (1.47)	11.34 (2.15)	13.37 (1.84)	8.93 (2.02)
Wilson *B*-factor	47.98	49.96	52.53	46.34	43.79	41.83
*R*_merge_	0.1273 (0.9111)	0.1581 (0.9594)	0.1150 (1.295)	0.08716 (0.9533)	0.07906 (0.9492)	0.1185 (1.002)
*R*_meas_	0.1400	0.1737	0.1257	0.0939	0.0851	0.1277
*CC*_1/2_^‡^	0.995 (0.587)	0.987 (0.514)	0.996 (0.501)	0.998 (0.681)	0.999 (0.762)	0.996 (0.687)
CC*	0.999 (0.860)	0.997 (0.824)	0.999 (0.817)	1.000 (0.900)	1.000 (0.930)	0.999 (0.902)
Reflections used for						
*R*_work_	0.1982 (0.2980)	0.1974 (0.3003)	0.1975 (0.3268)	0.2014 (0.2866)	0.1920 (0.2596)	0.1963 (0.2596)
*R*_free_	0.2334 (0.3574)	0.2361 (0.3498)	0.2379 (0.3777)	0.2286 (0.3265)	0.2327 (0.2704)	0.2395 (0.2975)
Number of non-hydrogen atoms	12460	12350	12352	12530	12519	12636
Macromolecules	12139	12139	12139	12127	12127	12127
Ligands	14	14	14	14	14	14
Water	307	197	199	389	378	495
Protein residues	1077	1077	1077	1075	1075	1075
RMSD^†^						
Bonds (Å)	0.003	0.004	0.003	0.003	0.007	0.004
Angles (^o^)	0.64	0.66	0.66	0.64	0.89	0.67
Ramachandran favored (%)	98.67	98.94	98.94	98.40	98.40	98.40
Ramachandran allowed (%)	1.06	0.93	0.79	1.33	1.33	1.33
Ramachandran outliers (%)	0.27	0.13	0.27	0.27	0.27	0.27
Clashscore	3.14	3.65	3.01	2.69	2.65	2.28
Average *B*-factor (Å^2^)	69.50	71.60	74.90	67.00	65.90	62.20
Macromolecules	69.90	72.00	75.30	67.50	66.30	62.80
Ligands	81.70	85.40	91.10	80.90	78.10	74.10
Solvent	51.00	51.20	53.60	51.90	50.80	48.50
PDB ID	5AV5	5AVB	5AVC	5AV6	5AV8	5AV9

^§^Values in parentheses are for highest resolution shell.

^‡^*CC_1/2_*, percentage of correlation between intensities from random half-datasets^60^.

^†^RMSD, root-mean-square deviation.

**Table 2:** Histone residues affected by the H4 tetra-acetylation and their interactions.

Histone (molecule)	Residue(s)	Neighbor residue involved in interaction	Interaction	DNA/histone (molecule)	Residue/nucleotide	Distance (Å)^§^
H3 (A)	F78	Q76	Inter-nucleosomal	Chain J	dC–62	2.94
H3 (E)	K37–P38		Inter-nucleosomal	Chain I	dC–5	7.36
H4 (B)	V21–N25, K31–P32		Inter-nucleosomal	Chain I	dT57	6.43
H4 (F)	H18		Inter-nucleosomal	H2A (C)	D90	3.02
H4 (F)	G101		Intra-nucleosomal	H4 (F)	M84	3.30
H2A (C)	K13		Inter-nucleosomal	Chain I	dC6	7.44
H2A (C)	P117–K118		Inter-nucleosomal	Chain J	dC34	13.08
H2A (G)	A14–K15		Inter-nucleosomal	Chain J	dA66	14.71
H2A (G)	A103		Intra-nucleosomal	H2A (G)	G105	3.16
H2B (D)	S32–E35		Intra-nucleosomal	Chain I	dG30	3.46
H2B (D)	S32–E35		Intra-nucleosomal	Chain J	dT50	3.34
H2B (D)	V48^‡^		Inter-nucleosomal	H3 (E)	D77^‡^	3.51^‡^
H2B (D)	V48		Inter-nucleosomal	H4 (F)	V21	3.37
H2B (H)	A124	S123	Inter-nucleosomal	Chain J	dT67	4.51

^§^Distance between the atoms involved in the interaction, or the nearest atoms between the indicated residue(s)/nucleotide.

^‡^Distance between the main-chain carbonyl O atom of V48 (molecule-D H2B) and the OD1 atom of D77 (molecule-E H3), which forms an inter-nucleosomal interaction in the crystal through Mn^++^. This inter-surface Mn^++^ is not significantly affected by the H4-tetra-acetylation.
