# Supplementary material for: Intra- and inter-nucleosomal interactions of the histone H4 tail revealed with a human nucleosome core particle with genetically-incorporated H4 tetra-acetylation
Source: Sci Rep. 2015 Nov 26;5:17204. doi: 10.1038/srep17204 (PMC4660432; doi:10.1038/srep17204)
Supplement: Supplementary Information [file srep17204-s1.pdf]

Supplementary Information

Intra- and inter-nucleosomal interactions of the histone H4 tail revealed with a human nucleosome core particle with genetically-incorporated H4 tetra-acetylation

Masatoshi Wakamori[1,2]*, Yoshifumi Fujii[1,3]*, Noriyuki Suka[1,4], Mikako Shirouzu[1,2], Kensaku Sakamoto[1,2], Takashi Umehara[1,2,5] & Shigeyuki Yokoyama[1,3]

[1]RIKEN Systems and Structural Biology Center, 1-7-22 Suehiro-cho, Tsurumi, Yokohama 230-0045, Japan, [2]RIKEN Center for Life Science Technologies, 1-7-22 Suehiro-cho, Tsurumi, Yokohama 230-0045, Japan, [3]RIKEN Structural Biology Laboratory, 1-7-22 Suehiro-cho, Tsurumi, Yokohama 230-0045, Japan, [4]School of Science and Engineering, Meisei University, 2-1-1 Hodokubo, Hino, Tokyo 191-8506, Japan, [5]PRESTO, Japan Science and Technology Agency (JST), 4-1-8 Honcho, Kawaguchi, Saitama 332-0012, Japan.

Correspondence and requests for materials should be addressed to S.Y. (yokoyama@riken.jp) or T.U. (takashi.umehara@riken.jp).

*These authors contributed equally to this work.

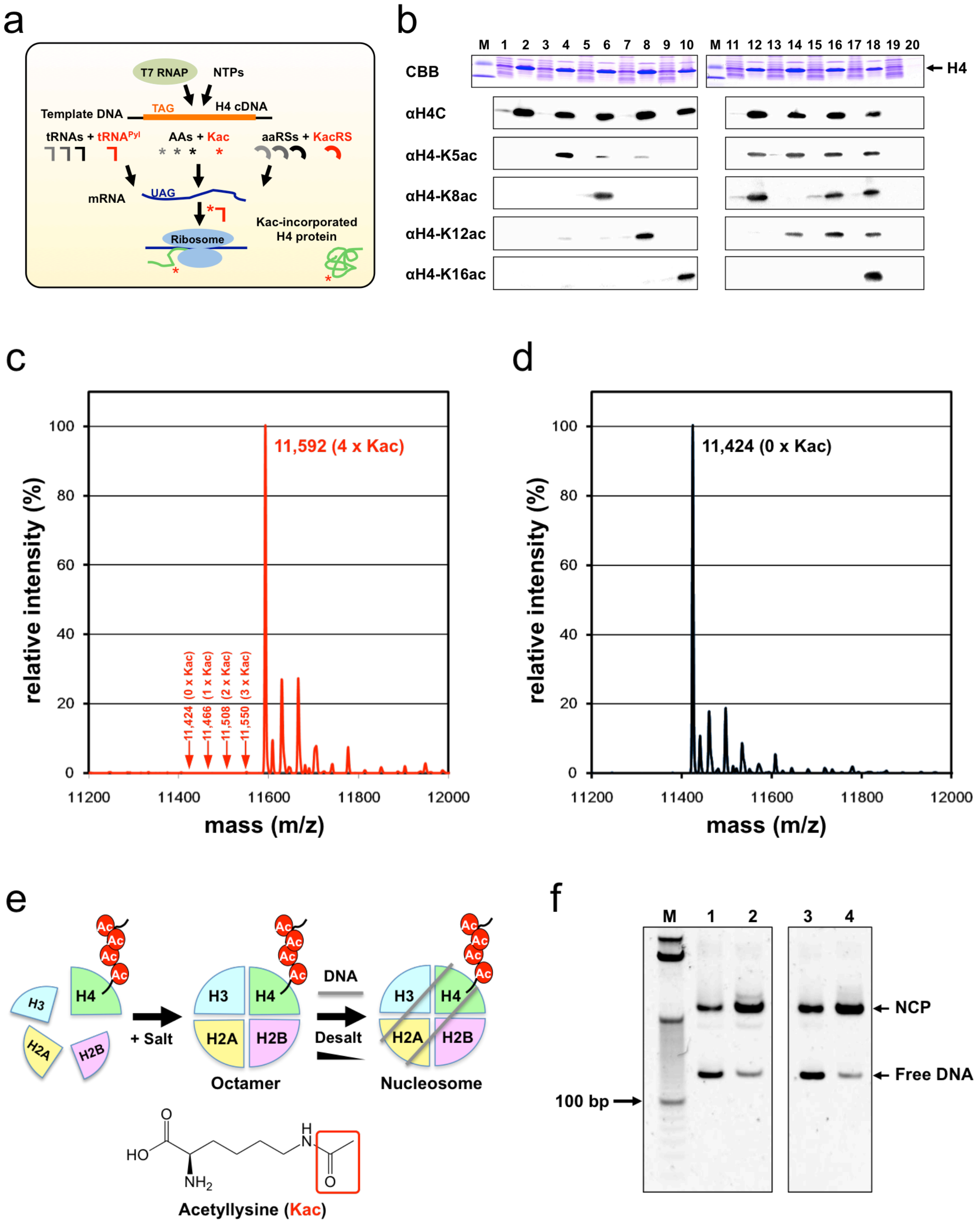

Supplementary Figure S1 | Reconstitution of acetyllysine-incorporated NCPs. (a) Schematic representation of the cell-free protein synthesis of acetyllysine-incorporated histone H4. Abbreviations are as follows: T7 RNAP, T7 RNA polymerase; NTPs, nucleotide triphosphates; $tRNA^{Pyl}$, pyrrolysine tRNA; AAs, amino acids; Kac, acetyllysine; aaRSs, aminoacyl-tRNA synthetases; and KacRS, engineered pyrrolysyl-tRNA synthetase specific to Kac. TAG in the

template DNA and UAG in the mRNA indicate the position at which Kac (shown by the red asterisk) is site-specifically incorporated during translation. (b) Synthesis and confirmation of acetyllysine-incorporated histone H4 proteins. The top panels are SDS-PAGE analyses with Coomassie Brilliant Blue staining (1 μl reaction solution), and the panels in the second to the bottom rows are western blots using antibodies recognizing the indicated histone H4 species (described below). M: protein size markers (top, 15 kDa; and bottom, 10 kDa). Odd- and even-numbered lanes indicate soluble and insoluble fractions of the cell-free protein synthesis solution after the reaction. Lanes 1 and 2: unmodified H4; lanes 3 and 4: K5-acetylated H4; lanes 5 and 6: K8-acetylated H4; lanes 7 and 8: K12-acetylated H4; lanes 9 and 10: K16-acetylated H4; lanes 11 and 12: K5/K8-di-acetylated H4; lanes 13 and 14: K5/K12-di-acetylated H4; lanes 15 and 16: K5/K8/K12-tri-acetylated H4; lanes 17 and 18: K5/K8/K12/K16-tetra-acetylated H4; lanes 19 and 20: no template DNA. Rabbit polyclonal antibodies used in the western blotting are: αH4C, anti-C-terminal region of H4 antibody (Abcam, ab10158); αH4-K5ac, anti-N-terminal tail of K5-acetylated H4 antibody (Millipore, 07-327); αH4-K8ac, anti-N-terminal tail of K8-acetylated H4 antibody (Millipore, 07-329); αH4-K12ac, anti-N-terminal tail of K12-acetylated H4 antibody (Millipore, 07-595); and αH4-K16ac, anti-N-terminal tail of K16-acetylated H4 antibody (Millipore, 07-328). (c, d) ESI mass spectrometric analyses of the synthesized H4 proteins. (c) N-terminal tag-cleaved K5/K8/K12/K16-tetra-acetylated H4 (red line). The predicted mass for the tetra-acetylated H4 protein is 11,592 Da. (d) N-terminal tag-cleaved unmodified H4 (black line). The predicted mass for the unmodified H4 protein is 11,424 Da. The difference of 168 Da between the two peaks corresponds to the addition of four acetyl groups. Note that there are no significant peaks detected at the masses of the non-, mono-, di-, and tri-acetylated H4 proteins in the tetra-acetylated H4 sample. (e) Schematic representation of the reconstitution of acetyllysine-incorporated NCPs. Synthesized H4 proteins with or without site-specific acetyllysine residue(s) were refolded and mixed with an equimolar amount of bacterially expressed histones H3, H2A, and H2B under high salt (2 M NaCl) conditions. The assembled histone octamers were then mixed with the nucleosomal DNA, and NCPs were reconstituted by gradient salt dialysis. The chemical structure of acetyllysine is shown at the bottom. The acetyl group is marked in red. (f) Confirmation of the formation of nucleosome core particles. Reconstituted NCPs analyzed by native PAGE. Lanes 1 and 2: unmodified NCP; lanes 3 and 4: NCP containing K5/K8/K12/K16-tetra-acetylated H4; lanes 1 and 3: samples after nucleosome reconstitution; and lanes 2 and 4: samples after purification through Mg^{++}-dependent precipitation. M: 10-bp DNA ladder marker.

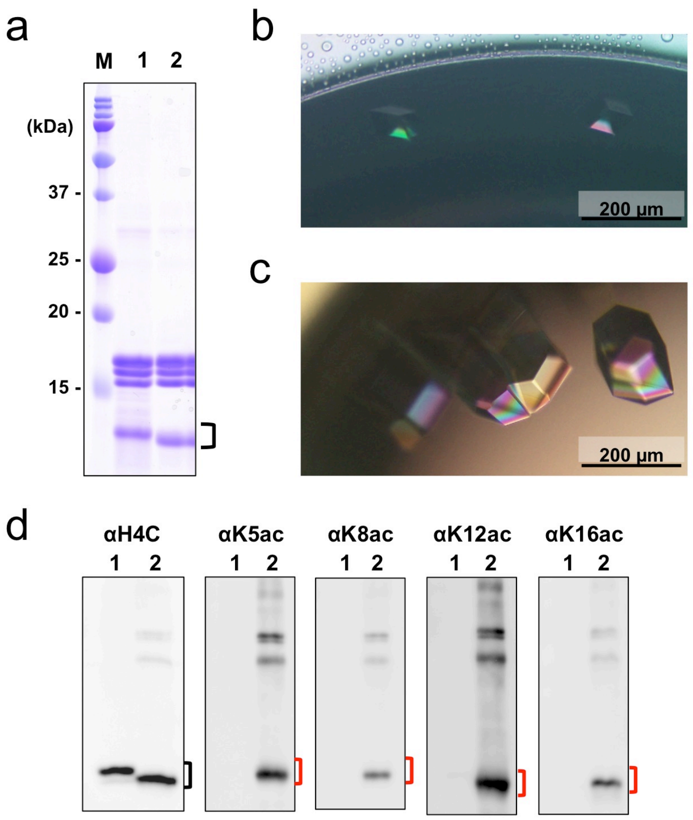

Supplementary Figure S2 | Crystallization of tetra-acetylated NCP. (a) SDS-PAGE profiles of NCPs used for crystallization. M: protein size markers (Bio-Rad, 161-0374); lane 1: NCP reconstituted with unmodified H4; and lane 2: NCP with K5/K8/K12/K16-tetra-acetylated H4. Note that the tetra-acetylated H4 protein migrates faster than the unmodified H4 protein, as marked by the bracket on the right. (b) Single crystals of NCP with H4-K5/K8/K12/K16-tetra-acetylated H4. (c) Single crystals of unmodified NCP. (d) Western blot analysis of the protein samples used for crystallization. Lane 1: sample collected from the drops incubated with NCP containing unmodified H4. Lane 2: sample of NCP containing K5/K8/K12/K16-tetra-acetylated H4. SDS-PAGE gel-transferred membranes were immunoblotted with the indicated residue-specific acetyllysine recognition antibodies: αH4C, anti-C-terminal region of H4 antibody (Abcam, ab10158); αH4-K5ac, anti-N-terminal tail of K5-acetylated H4 antibody (Millipore, 07-327); αH4-K8ac, anti-N-terminal tail of K8-acetylated H4 antibody (Millipore, 07-329); αH4-K12ac, anti-N-terminal tail of K12-acetylated H4 antibody (Millipore, 07-595); and αH4-K16ac, anti-N-terminal tail of K16-acetylated H4 antibody (Millipore, 07-328). The positions of the H4 proteins are indicated by the brackets on the right.

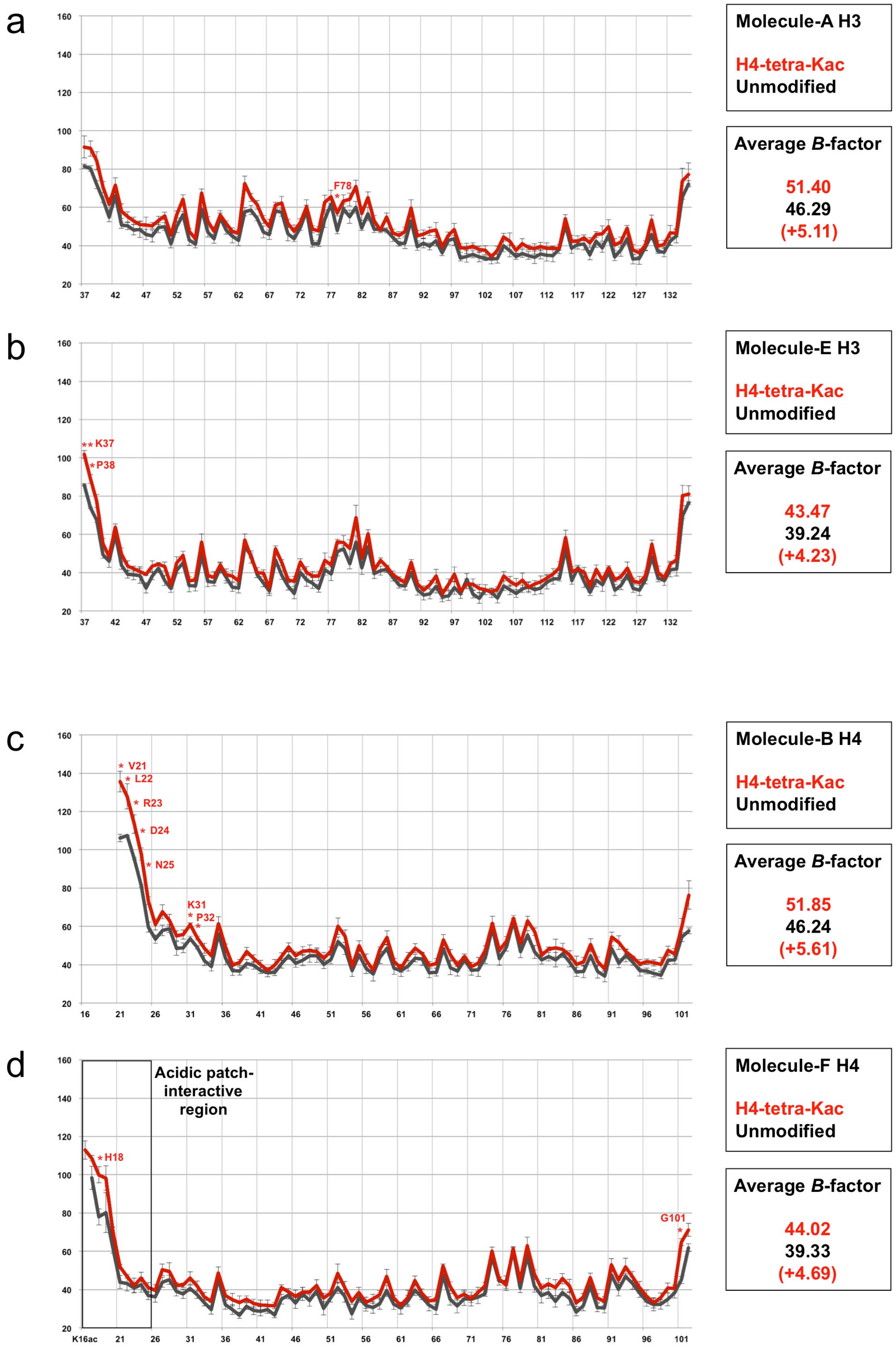
a
Molecule-A H3
H4-tetra-Kac
Unmodified
Average B-factor
51.40
46.29
(+5.11)
F78
b
Molecule-E H3
H4-tetra-Kac
Unmodified
Average B-factor
43.47
39.24
(+4.23)
** K37
* P38
c
Molecule-B H4
H4-tetra-Kac
Unmodified
Average B-factor
51.85
46.24
(+5.61)
* V21
* L22
* R23
* D24
* N25
K31
* P32
d
Molecule-F H4
H4-tetra-Kac
Unmodified
Average B-factor
44.02
39.33
(+4.69)
Acidic patch-interactive region
* H18
G101
K16ac

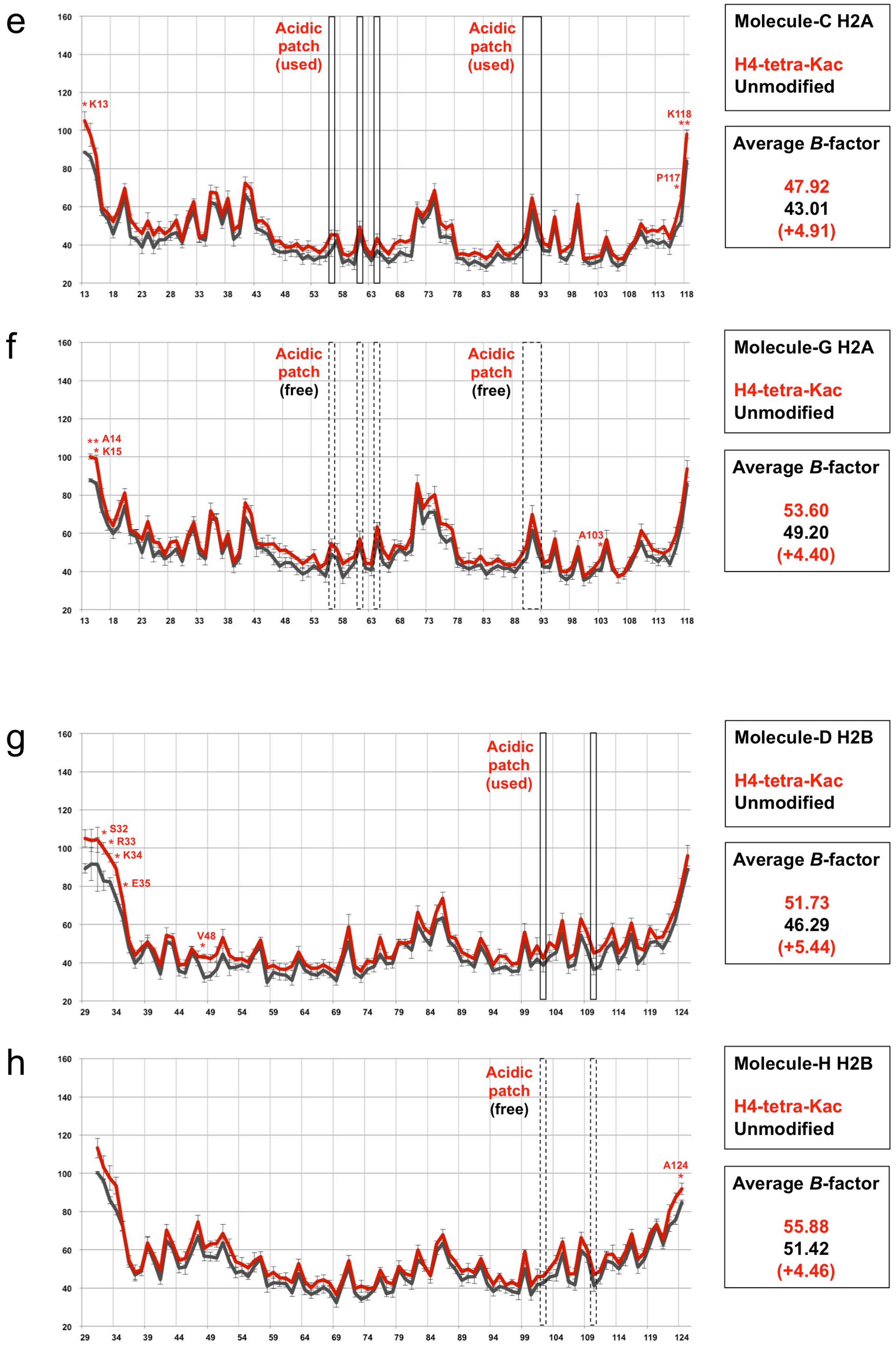
e
Acidic patch (used)
Acidic patch (used)
* K13
K118 **
P117 *
Molecule-C H2A
H4-tetra-Kac
Unmodified
Average B-factor
47.92
43.01
(+4.91)
f
Acidic patch (free)
Acidic patch (free)
** A14
* K15
A103 *
Molecule-G H2A
H4-tetra-Kac
Unmodified
Average B-factor
53.60
49.20
(+4.40)
g
Acidic patch (used)
* S32
* R33
* K34
* E35
V48 *
Molecule-D H2B
H4-tetra-Kac
Unmodified
Average B-factor
51.73
46.29
(+5.44)
h
Acidic patch (free)
A124 *
Molecule-H H2B
H4-tetra-Kac
Unmodified
Average B-factor
55.88
51.42
(+4.46)

Supplementary Figure S3 | *B*-factor plots of the H4-tetra-acetylated NCP histones. (a) Molecule-A H3, (b) Molecule-E H3, (c) Molecule-B H4, (d) Molecule-F H4, (e) Molecule-C H2A, (f) Molecule-G H2A, (g) Molecule-D H2B, and (h) Molecule-H H2B. The X-axis indicates the position of the amino acid residue, and the Y-axis indicates the *B*-factor ($Å^2$) over all modeled atoms of the amino acid. In each panel, the average of the *B*-factors from three independently refined structures of H4-tetra-acetylated (red) and unmodified (black) NCPs is respectively presented as mean ± SD for each residue. Asterisks: $^*P < 0.05$ and $^{**}P < 0.01$ in two-tailed Student's *t*-test. The average scores of the *B*-factors of each histone molecule are shown in the boxes on the right, as follows: average score of H4-tetra-acetylated NCP (top, red), average score of unmodified NCP (middle, black), and difference in the average scores (bottom, red in parentheses). In (d), the *B*-factor of the residue K16ac in H4-tetra-acetylated NCP is omitted for the calculation of the average score, for comparison. In (e) – (h), the positions of acidic patch residues are marked by solid (molecules C and D) and dashed (molecules G and H) rectangles, respectively. Note that only the acidic patch in molecules C and D (H2A/H2B) interacts with the molecule-F H4 tail of the symmetrically-related NCP in the crystal.
